# Racial and Ethnic Disparities in Clinical Characteristics and Outcomes in Adults With Encephalitis: A Retrospective Study

**DOI:** 10.1177/11795735251414833

**Published:** 2026-01-15

**Authors:** Sienna Wu, Rodrigo Hasbun, Ralph Habis, Jordan Benderoth, Ivany Patel, Ashutosh Gupta, Megan Goyal, Arun Venkatesan, John C. Probasco, Paris Bean, Ashley Heck, Laya Rao, Rajesh K. Gupta

**Affiliations:** 1 Department of Neurology, McGovern Medical School, UTHealth Science Center, Houston, TX, USA; 2 Department of Medicine, Section of Infectious Disease, McGovern Medical School, UTHealth Science Center, Houston, TX, USA; 3 Johns Hopkins Encephalitis Center, Johns Hopkins University, Baltimore, MD, USA

**Keywords:** racial disparities, encephalitis, infectious encephalitis, autoimmune encephalitis, socioeconomic disparities

## Abstract

**Background and Objectives:**

This study aims to assess whether adult patients with encephalitis from different racial and ethnic backgrounds exhibit significant differences in clinical presentation, diagnostic findings, and outcomes.

**Design and Methods:**

A retrospective cohort study was conducted by utilizing the electronic health records of encephalitis patients in the greater Houston and Baltimore areas. Patients were categorized by race/ethnicity into White or ethnic minority (including Black, Hispanic, and Asian patients). Data was analyzed for the presence of significant differences in clinical characteristics between the two groups.

**Results:**

Among 599 patients, 312 (52.1%) were White and 287 (47.9%) were of an ethnic minority. White patients were more often over sixty years-old upon presentation (43.1% vs 23.9%, *P* < 0.001) and more likely to present with memory deficits (36% vs 26.3%, *P* = 0.012). Ethnic minority patients more frequently presented with co-existing HIV (20.3% vs 3.4%, *P* < 0.001), severe organ dysfunction (44% vs 34.4%, *P* = 0.028), cerebrospinal fluid (CSF) pleocytosis (white blood cell count ≥5 cells/µL) (83.1% vs 69.3%, *P* < 0.001), and abnormal electroencephalogram (EEG) findings (84.3% vs 71.9%, *P* = 0.035). Ethnic minority patients also had worse outcomes on the Glasgow Outcome Scale (GOS) as defined by GOS <4 (59.3% vs 47.2%, *P* = 0.005). Binary logistic regression identified abnormal magnetic resonance imaging (MRI) and Glasgow Coma Scale (GCS) <13 as independent predictors of an adverse clinical outcome (GOS <4) with an adjusted odds ratio [95% confidence interval] (*P* value) of 1.609 [1.042-2.486] (*P* = 0.032) and 2.689 [1.675-4.317] (*P* < .001), respectively.

**Conclusion:**

Ethnic minority patients with encephalitis present at a younger age and are more likely to have co-existing HIV, severe initial organ dysfunction, CSF pleocytosis, abnormal EEG findings, and worse clinical outcomes. Abnormal MRI and GCS <13 are independent predictors of an unfavorable clinical outcome and may aid in risk stratification.

## Plain Language Summary

Race and ethnicity have been shown to affect patient symptoms and outcomes in various diseases, including autoimmune neurological diseases. There has not yet been a comprehensive study on how race and ethnicity affects all causes of encephalitis in adults, including both infectious and autoimmune sources. Our research team used patient records from the past and from two major cities to collect data on patient demographics, symptoms, diagnostic and imaging findings, treatment received while hospitalized, and outcomes/recovery. Patients were categorized by race/ethnicity into two groups for this initial study; White or ethnic minority (including Black, Hispanic, and Asian patients). We analyzed the data to see if there were major differences in any factors between the two groups. The total number of patients was 599, with 52.1% being White and 47.9% of an ethnic minority. We found that White patients tended to present at a much older age and with memory gaps. Ethnic minority patients were sicker when they presented to the hospital in terms of other diseases they co-presented with, as well as initial imaging and lab findings. Our ethnic minority patients had worse outcomes in terms of neurological recovery and functional status by their discharge. A separate analysis showed that abnormal magnetic resonance imaging (MRI) results and a certain level of consciousness upon admission were indicative of worse outcomes by discharge. Our study revealed that ethnic minority patients with encephalitis are more likely to present younger and with more severe illness upon hospitalization, which may be a potential reason for worse functional outcomes. More work needs to be done to differentiate symptoms and outcomes between the two groups based on the cause of encephalitis (infectious vs. autoimmune). This study has also identified abnormal MRI and level of consciousness upon admission as potential predictors of worse outcomes, which should be further studied.

## Introduction

Encephalitis is an inflammation of the brain caused by an infectious or autoimmune etiology that can induce neurological and psychiatric symptoms, and if not treated immediately, can lead to serious complications such as brain injury or even death. It is estimated to occur in around 5-10 per 100,000 U.S. persons each year^1^ with an average inpatient mortality rate of 5.6%,^2,3^ demonstrating a considerable burden of disease in the U.S.

The influence of racial disparities in autoimmune disease outcomes, such as multiple sclerosis (MS), has been established in the current literature.^4-6^ Though MS has a higher incidence in White people, recent research has demonstrated that ethnic minority populations, most notably African American and Hispanics, are more likely to develop worse outcomes and be negatively impacted by social determinants of health, further exacerbating differences in outcomes.^6^ Research in the past decade on autoimmune encephalitis (AE) has demonstrated that non-White race is positively associated with anti-dopamine receptor 2 encephalitis incidence in pediatric patients.^7^ Data has also suggested that non-White race is a risk factor for anti-N-methyl-D-aspartate receptor (NMDAR) encephalitis,^8,9^ with African Americans being disproportionately affected in prevalence and incidence.^10^ A retrospective study in 2013 on encephalitis found that Asian/Pacific Islander patients were less likely to recover when compared to patients of other races, though the study was limited to pediatric populations.^11^ No previous studies have examined the association between racial and ethnic disparities, and clinical characteristics and outcomes in all causes of adult encephalitis.

The goal of this retrospective study was to assess racial and ethnic disparities across all causes of adult encephalitis with regards to clinical characteristics, diagnostic and imaging findings, and clinical outcomes. Investigating these relationships may reveal whether certain populations experience poorer clinical prognoses and offer insight into improving outcomes for encephalitis patients. To address these objectives, we conducted a multicenter retrospective cohort study to evaluate racial disparities in adult encephalitis and identify clinical factors associated with adverse patient outcomes. Given the limited literature on racial and ethnic disparities in encephalitis, this study initially examined all-cause encephalitis and broadly categorized patients by race/ethnicity into White or ethnic minority to identify overarching patterns.

## Materials and Methods

### Standard Protocol Approvals, Registrations, and Patient Consents

This study was approved by the Institutional Review Boards at the University of Texas and Johns Hopkins Hospital. This was designed as a retrospective study in which adult encephalitis patients were identified from electronic health record databases from medical centers in the Greater Houston and Greater Baltimore regions between January 2005 to December 2022.

### Inclusion Criteria

Patients were identified using the International Classification of Disease (ICD-9) diagnosis codes. Inclusion criteria was determined with the 2013 International Encephalitis Consortium (IEC) criteria.^12^ For initial analysis, patients were dichotomized by race/ethnicity into White or ethnic minority (including Black, Hispanic, and Asian patients) to identify broad disparities, with plans for more granular stratification in future work.

### Data Collection

Data was collected from patient charts, with most variables obtained from the initial patient encounter at the emergency department, including demographics, comorbidities, presenting signs and symptoms, laboratory values, and initial imaging results. The Charlson Comorbidity Index (CCI) was scored using previous medical history, with a score greater than 2 indicating the presence of comorbidity.^13^ Immune status was also determined based on several factors (presence of human immunodeficiency virus (HIV), recent chemotherapy within 1 month, solid organ or bone marrow transplantation, having received ≥20 mg of prednisone or equivalent for >1 month, or congenital immunodeficiency). Several scores were calculated to assess consciousness and severity of brain injury and illness upon presentation. Glasgow Coma Scale (GCS) and Full Outcome of Responsiveness (FOUR) Score were utilized for levels of consciousness.^14,15^ The Sequential Organ Failure Assessment (SOFA) was utilized to determine organ function/failure at presentation and predict intensive care unit (ICU) mortality.^16^ Etiology was defined as infectious, seropositive autoimmune, or unknown.

Clinical outcomes were primarily defined by the Glasgow Outcome Scale (GOS), which measures patient recovery following traumatic brain injury.^17^ A score of 5 indicates good recovery with minor neurological and psychological deficits, with the rest of the scale ranging from moderate disability with functional independence (4) to death (1). Our study classified GOS <4 as an adverse clinical outcome.^18,19^ Other outcome measures used were length of stay (in days), ICU admission, and mortality.

### Statistical Analysis

Data analysis was conducted using IBM Statistical Package for the Social Sciences version 29.0.0. Bivariate analyses of dichotomous clinical variables were performed using the Pearson Chi-Square test or Fisher’s Exact test, as appropriate. For continuous variables, the median and interquartile range (IQR) were reported, and the Mann-Whitney U test was used to compare medians. Variables with a *P*-value less than 0.05 and a 95 percent confidence interval were considered statistically significant. The subgroup analysis utilized the same statistical approach as the primary analysis.

A separate bivariate analysis was performed to determine variables associated with adverse clinical outcomes, as defined by GOS <4. Variables significantly associated with an adverse clinical outcome were entered into a binary logistic regression analysis, validated by bootstrapping and the Hosmer-Lemeshow test, with 95% confidence intervals.

A socioeconomic factors analysis was performed. Insurance status was recorded from patient charts and defined as public, private, dual (public and private), or none. Median household income for each patient was determined based on zip code at time of admission using the Kinder Institute for Urban Research Houston Community Data Connections Database and U.S. Census Data.

Most variables in the combined database were present in all patients. Patients who did not have data for a certain variable were excluded from the analysis of the variable.

## Results

### Patient Demographics and Etiology

Of 647 patients who met the IEC criteria for encephalitis, 599 had an identified race and were included in this study. 312 were White (52.1%) and 287 were of an ethnic minority (47.9%). The median age [IQR] was 56 [41.25-67] for the White population and 44 [30-59] for the ethnic minority population (*P* < 0.001). A higher proportion of the White population was older than 60 years upon presentation (43.1% vs 23.9%, *P* < 0.001). The prevalence of females affected with encephalitis was not significantly different between the White and ethnic minority populations (49% vs 51.6%, *P* = 0.536). No significant differences were observed between the two groups in Charlson Comorbidity Index (CCI) > 2, or presence of comorbidities (50.3% vs 43.2%, *P* = 0.081), or immunocompromised status (20.5% vs 26.5%, *P* = 0.085). A higher proportion of ethnic minority patients had coexisting HIV infection (20.3% vs 3.4%, *P* < 0.001) (Table 1).

**Table 1.** Demographics, Etiology, and Clinical Features Between White and Ethnic Minority Populations in Adult Encephalitis

Clinical feature	White	Ethnic minority	*P* value^a^
n/N (%)	312/599 (52.1)	287/599 (47.9)	
Age, median (IQR)	56 (41.25-67)	44 (30-59)	<.001*
Age >60 years	134/311 (43.1)	68/285 (23.9)	<.001*
Female sex	153/312 (49)	148/287 (51.6)	0.536
Coexisting medical conditions			
Charlson comorbidity index >2	157/312 (50.3)	124/287 (43.2)	0.081
HIV	10/293 (3.4)	56/276 (20.3)	<.001*
Immunocompromised^b^	64/312 (20.5)	76/287 (26.5)	0.085
Etiology			
Infectious	132/312 (42.3)	133/287 (46.3)	0.321
Autoimmune (seropositive)	49/312 (15.7)	53/287 (18.5)	0.369
Unknown	131/312 (42)	101/287 (35.2)	0.088
Presenting symptoms			
Acute presentation (<5 days)	142/285 (49.8)	153/283 (54.1)	0.312
Fever	154/289 (53.3)	154/279 (55.2)	0.648
Focal neurological deficits	126/306 (41.2)	99/287 (34.5)	0.094
Seizures	118/296 (39.9)	113/283 (39.9)	0.987
Headache	137/264 (51.9)	136/255 (53.3)	0.743
Neck stiffness	25/116 (21.6)	30/191 (15.7)	0.195
Nausea	46/119 (38.7)	74/199 (37.2)	0.794
Photophobia	13/103 (12.6)	24/169 (14.2)	0.712
Memory deficits	109/303 (36)	75/285 (26.3)	0.012*
Movement disorder	11/135 (8.1)	25/223 (11.2)	0.350
Presenting signs			
GCS, median (IQR)	14 (10-15)	14 (12-15)	0.089
GCS <13	107/312 (34.3)	65/239 (27.2)	0.075
Hypotension^c^	15/279 (5.4)	11/276 (4)	0.438
SOFA >3	87/253 (34.4)	109/248 (44)	0.028*
FOUR score <14	82/299 (27.4)	50/236 (21.2)	0.097

Abbreviations: HIV, Human Immunodeficiency Virus; GCS, Glasgow Coma Scale; SOFA, Sequential Organ Failure Assessment; FOUR Score, Full Outcome of Responsiveness Score.

*Significant value as defined by *P* < 0.05.

^a^*P*-value for difference between White and ethnic minority patients.

^b^Immunocompromised is defined as having Human immunodeficiency virus (HIV), recent chemotherapy (<1 month ago), solid organ or bone marrow transplantation, receiving ≥ 20 mg of prednisone or the equivalent for >1 month, or congenital immunodeficiency.

^c^Defined as a mean arterial pressure <70 mmHg.

Both populations had similar proportions of patients with an infectious, seropositive autoimmune, and unknown etiology (Table 1).

### Onset and Clinical Presentation

The proportion of patients presenting acutely (within <5 days) did not differ significantly between the two groups (49.8% vs 54.1%, *P* = 0.312). More White patients presented with memory deficits (36% vs 26.3%, *P* = 0.012). Presence of fever, focal neurological deficits, seizures, headache, neck stiffness, nausea, photophobia, and movement disorder were comparable between the two groups. Both populations had a median GCS score of 14 upon presentation and a similar proportion of patients presenting with a FOUR Score <14 (27.4% vs 21.2%, *P* = 0.097). More ethnic minority patients presented with a SOFA score >3, indicating severe organ dysfunction and/or failure upon presentation (44% vs 34.4%, *P* = 0.028) (Table 1).

### Serum and Imaging Findings

Ethnic minority patients were more likely to present with CSF pleocytosis (white blood cell count ≥5 cells/µL) than White patients (83.1% vs 69.3%, *P* < .001). A greater proportion of ethnic minority patients had abnormal EEG findings than their White counterparts (84.3% vs 71.9%, *P* = 0.003). No other significant differences between the two patient populations were noted in the CSF analysis or other imaging modalities (Table 2).

**Table 2.** Diagnostic Findings, Treatment, and Outcomes Between White and Ethnic Minority Populations in Adult Encephalitis

Clinical feature	White	Ethnic minority	*P* value^a^
n/N (%)	312/599 (52.1)	287/599 (47.9)	
CSF analysis			
Opening pressure, median (IQR)	20 (17-27)	18.5 (15-28)	0.922
CSF white blood cell count, median (IQR)	27 (12-244)	27.5 (13-180)	0.972
CSF pleocytosis^b^, n/N (%)	205/296 (69.3)	236/284 (83.1)	<.001*
CSF neutrophil, median (IQR)	1 (0-17)	6 (0-39)	0.113
CSF protein, median (IQR)	76 (61-149)	68 (51-124.6)	0.810
CSF glucose, median (IQR)	61 (47-79)	60.5 (44-66)	0.607
CSF glucose <45, n/N (%)	46/287 (16)	61/282 (21.6)	0.087
Lack of inflammatory CSF^c^, n/N (%)	71/292 (24.3)	75/284 (26.4)	0.580
Imaging and testing, n/N (%)			
Abnormal CT findings	37/118 (31.4)	69/201 (34.3)	0.586
Abnormal MRI findings	142/256 (55.6)	138/232 (60)	0.371
Cerebral edema	40/310 (12.9)	33/284 (11.6)	0.634
Abnormal EEG findings	159/221 (71.9)	150/178 (84.3)	0.003*
Treatment, n/N (%)			
Vancomycin	144/305 (47.2)	176/286 (61.5)	<.001*
Acyclovir	186/303 (61.4)	184/286 (64.3)	0.459
Steroids	186/304 (61.2)	160/286 (55.9)	0.196
PLEX	43/306 (14.1)	41/287 (14.3)	0.935
IVIG	59/306 (19.3)	43/287 (15)	0.166
Rituximab	20/305 (6.6)	30/287 (10.5)	0.088
Cyclophosphamide	8/305 (2.6)	10/287 (3.5)	0.542
Outcomes, n/N (%)			
ICU admission	113/304 (37.2)	117/286 (40.9)	0.352
Length of stay in days, median (IQR)	11 (6-18)	12 (7-30)	0.009*
GOS <4	144/305 (47.2)	144/243 (59.3)	0.005*
Mortality	26/312 (8.3)	18/287 (6.3)	0.334

Abbreviations: CSF, Cerebrospinal Fluid; CT, Computed Tomography; MRI, Magnetic Resonance Imaging; EEG, Electroencephalogram; PLEX, Plasmapheresis; IVIG, Intravenous Immunoglobulin; ICU, Intensive Care Unit; GOS; Glasgow Outcome Scale.

*Significant value as defined by *P* < 0.05.

^a^*P*-value for difference between White and ethnic minority patients.

^b^White blood cell count ≥5 cells/µL.

^c^Meets both criteria of protein count <50 and white blood cell count <50.

### Treatment

A higher proportion of ethnic minority patients were treated with vancomycin compared to the White population (61.5% vs 47.2%, *P* < 0.001). No meaningful differences were found regarding the use of antivirals, steroids, and immunotherapy (Table 2).

### Clinical Outcomes

A higher proportion of ethnic minority patients had adverse clinical outcomes (59.3% GOS <4 vs 47.2%, *P* = 0.005). In addition, ethnic minority patients had a greater median [IQR] length of hospitalization (12 [7-30] vs 11 [6-18], *P* = 0.009) (Table 2).

An additional bivariate analysis was performed to determine variables significantly associated with an adverse clinical outcome of GOS <4 (Table 3). Six significant variables from this separate bivariate analysis (age >60 years, ethnic minority race, Charlson Comorbidity Index >2, headache, GCS <13, and abnormal magnetic resonance imaging (MRI)) were incorporated into a multivariate logistic regression. The regression revealed that abnormal MRI and GCS <13 were independent predictors of an adverse clinical outcome in adult encephalitis, with an adjusted odds ratio [95% confidence interval] (*P* value) of 1.609 [1.042-2.486] (*P* = 0.032) and 2.689 [1.675-4.317] (*P* < .001), respectively.

**Table 3.** Logistic Regression and Bivariate Factors Associated With an Adverse Clinical Outcome, Defined as Glasgow Outcome Scale <4

Clinical feature	Bivariate analysis	Logistic regression^c^
	Adjusted odds ratio (95% CI^a^) *P* value^b^	
Demographics/comorbidities		
Age >60 years	0.591 (0.417-0.838) 0.003	0.657 (0.380-1.136) 0.133
Female sex	1.262 (0.909-1.753) 0.164	
Ethnic minority race	1.626 (1.157-2.286) 0.005	1.438 (0.922-2.242) 0.109
Immunocompromised^d^	1.038 (0.701-1.535) 0.854	
HIV	1.101 (0.637-1.904) 0.729	
Charlson comorbidity index >2	0.667 (0.480-0.928) 0.016	0.976 (0.588-1.617) 0.924
Presenting signs/symptoms		
Acute presentation	0.957 (0.683-1.341) 0.797	
Fever	1.011 (0.721-1.417) 0.951	
Focal neurological deficits	1.113 (0.793-1.562) 0.534	
Seizures	0.791 (0.564-1.110) 0.175	
Headache	1.555 (1.091-2.217) 0.014	1.419 (0.915-2.200) 0.118
Memory deficits	1.004 (0.705-1.430) 0.980	
Movement disorder	1.003 (0.484-2.080) 0.993	
Hypotension^e^	0.697 (0.311-1.566) 0.380	
SOFA >3	0.734 (0.507-1.062) 0.100	
GCS <13	2.721 (1.859-3.983) <.001	2.689 (1.675-4.317) <.001
Etiology		
Infectious	0.996 (0.716-1.384) 0.979	
Autoimmune (seropositive)	0.940 (0.617-1.434) 0.775	
Unknown	1.046 (0.743-1.471) 0.797	
Diagnostic findings		
CSF Pleocytosis^f^	0.945 (0.645-1.384) 0.772	
CSF Glucose <45 mg/dl	1.200 (0.773-1.862) 0.416	
Cerebral edema	0.752 (0.452-1.249) 0.269	
Abnormal CT	0.817 (0.499-1.338) 0.421	
Abnormal MRI	1.541 (1.068-2.223) 0.021	1.609 (1.042-2.486) 0.032
Abnormal EEG	0.968 (0.603-1.555) 0.893	
Treatment		
Vancomycin	1.074 (0.772-1.496) 0.670	
Acyclovir	0.973 (0.690-1.371) 0.876	
Steroids	0.777 (0.555-1.089) 0.142	
PLEX	0.690 (0.438-1.088) 0.109	
IVIG	0.812 (0.531-1.240) 0.334	
Rituximab	1.088 (0.616-1.919) 0.772	
Cytoxan	0.558 (0.213-1.461) 0.229	
ICU Admission	0.862 (0.615-1.208) 0.387	

Abbreviations: HIV, Human Immunodeficiency Virus; SOFA, Sequential Organ Failure Assessment; GCS, Glasgow Coma Scale; CSF, Cerebrospinal Fluid; CT, Computed Tomography; MRI, Magnetic Resonance Imaging; EEG, Electroencephalogram; PLEX, Plasmapheresis; IVIG, Intravenous Immunoglobulin; ICU, Intensive Care Unit.

^a^Confidence Interval.

^b^*p*-value for difference between White and ethnic minority patients.

^c^Clinical features that were significant in the bivariate analysis, as defined by *P <* 0.05, were entered into a logistic regression. This included Age >60 years, Ethnic Minority Race, Charlson Comorbidity Index >2, Headache, GCS <13, and Abnormal MRI. Hosmer-Lemeshow test was not significant (*P =* 0.787). All variables were validated internally with bootstrap (*P < 0.05*).

^d^Immunocompromised is defined as having Human immunodeficiency virus (HIV), recent chemotherapy (<1 month ago), solid organ or bone marrow transplantation, receiving ≥ 20 mg of prednisone or the equivalent for >1 month, or congenital immunodeficiency.

^e^Defined as a mean arterial pressure <70 mmHg.

^f^White blood cell count ≥5 cells/µL.

### Socioeconomic Status

**Figure 1.**
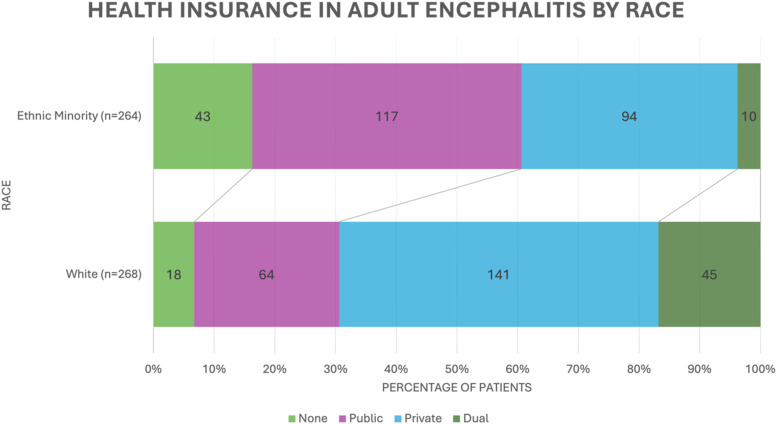
Health insurance in adult encephalitis by race

Our study also aimed to identify the role of socioeconomic status in the clinical characteristics and outcomes of patients with encephalitis using health insurance type and median household income level. 569 patients in our database had documented health insurance, with 188 (33%) on public plans, 259 (45.5%) on private, 59 (10.4%) on dual coverage (having both public and private plans), and 63 (11.1%) uninsured (Table 4) (see Figure 1 for health insurance type by race).

**Table 4.** Socioeconomic Factors Analysis by Health Insurance Status in Adult Encephalitis

Clinical feature	None	Public	Private	Dual^a^	*P* value^b^
n/N (%)	63/569 (11.1)	188/569 (33)	259/569 (45.5)	59/569 (10.4)	
Age >60 years	8/63 (12.7)	83/187 (44.4)	67/259 (25.9)	45/59 (76.3)	<.001*
Ethnic minority race	43/61 (70.5)	117/181 (64.6)	94/235 (40)	10/55 (18.2)	<.001*
Immunocompromised	16/63 (25.4)	58/188 (30.9)	45/259 (17.4)	10/59 (16.9)	0.005*
HIV positive	14/61 (23)	38/177 (21.5)	9/242 (3.7)	1/59 (1.7)	<.001*
CCI >2	21/63 (33.3)	111/188 (59)	87/259 (33.6)	49/59 (83.1)	<.001*
Infectious	32/63 (50.8)	98/188 (52.1)	100/259 (38.6)	21/59 (35.6)	0.012*
Autoimmune	6/63 (9.5)	22/188 (11.7)	57/259 (22)	16/59 (27.1)	0.002*
ICU admission	30/63 (47.6)	82/187 (43.9)	96/257 (37.4)	24/57 (42.1)	0.361
Mortality	4/63 (6.3)	16/188 (8.5)	15/259 (5.8)	3/59 (5.1)	0.661
GOS <4	34/54 (63)	91/167 (54.5)	109/226 (48.2)	25/59 (42.4)	0.096

Abbreviations: HIV, Human Immunodeficiency Virus; CCI, Charlson Comorbidity Index; ICU, Intensive Care Unit; GOS, Glasgow Outcome Scale.

*Significant value as defined by *P* < 0.05.

^a^Dual coverage indicates having both private and public insurance plans.

^b^*p*-value for difference between different insurance plans.

Patients with dual coverage were more likely to be > 60 years old (dual 76.3% vs none 12.7% vs public 44.4% vs private 25.9%, *P* < 0.001) and have a CCI >2 (dual 83.1% vs none 33.3% vs public 59% vs private 33.6%, *P* < 0.001) compared to the other three insurance groups. Ethnic minority patients were more likely to be uninsured or on public insurance (none 70.5% vs public 64.6% vs private 40% vs dual 18.2%, *P* < 0.001). A greater percentage of patients with no insurance or with public insurance were immunocompromised (none 25.4% vs public 30.9% vs private 17.4% vs dual 16.9%, *P* = 0.005) and HIV-positive compared to those with private or dual coverage (none 23% vs public 21.5% vs private 3.7% vs dual 1.7%, *P* < 0.001). Lack of insurance and public insurance were associated with infectious etiology (none 50.8% vs public 52.1% vs private 38.6% vs dual 35.6%, *P* = 0.012), while dual coverage was associated with autoimmune etiology (dual 27.1% vs none 9.5% vs public 11.7% vs private 38.6%, *P* = 0.002). Lower median household income was associated with being an ethnic minority (59,224 vs 90,772, *P* < 0.001), immunocompromised (68,962 vs 80,335, *P* = 0.001), HIV-positive (50,284 vs 80,692, *P* < 0.001), and an infectious etiology (71,609 vs 80,122, *P* < 0.001) (Supplemental Table 1). ICU admission, mortality, and GOS <4 had no significant differences between insurance groups or income levels.

### Etiology Subgroup Analysis

Infectious encephalitis and autoimmune encephalitis can often present distinctly, with each set of etiologies having their own clinical features that allow for comparison.^20^ Thus, we conducted a series of subgroup analyses to determine whether there were differences between White and ethnic minority patients within infectious and autoimmune etiologies (Table 5).

**Table 5.** Infectious and Autoimmune Etiology Subgroups in Adult Encephalitis, Significant Variables

Clinical feature			
n/N (%)	White	Ethnic minority	P value^a^
Infectious (n = 265)			
Age >60 years	59/125 (47.2)	41/146 (28.1)	0.001
Presence of HIV	5/111 (4.5)	39/137 (28.5)	<.001
Focal neurological deficits	52/125 (41.6)	43/147 (29.3)	0.033
Cerebral edema	22/124 (17.7)	13/146 (8.9)	0.031
Acyclovir	103/125 (82.4)	101/147 (68.7)	0.009
ICU Admission	54/123 (43.9)	44/146 (30.1)	0.019
Autoimmune, seropositive (n = 102)			
Age >60 years	24/48 (50)	7/53 (13.2)	<.001
Female sex	20/48 (41.7)	36/53 (67.9)	0.011
Charlson comorbidity index >2	26/48 (54.2)	9/53 (17)	<.001
Focal neurological deficits	25/48 (52.1)	16/53 (30.2)	0.021
CSF pleocytosis^b^	20/45 (44.4)	40/53 (75.5)	0.002
Vancomycin	8/47 (17)	20/53 (37.7)	0.025
Rituximab	9/47 (19.1)	26/53 (49.1)	0.002
ICU admission	11/47 (23.4)	26/53 (49.1)	0.010
Length of stay in days, median (IQR)	11 (6-23)	33 (17-54)	<.001

Abbreviations: HIV, Human Immunodeficiency Virus; ICU, Intensive Care Unit; CSF, Cerebrospinal Fluid.

^a^P-value for difference between White and ethnic minority patients.

^b^White blood cell count ≥5 cells/µL.

#### Infectious Subgroup

In this subgroup, more White patients presented >60 years old (47.2% vs 28.1%, *P* = 0.001) with focal neurological deficits (41.6% vs 29.3%, *P* = 0.033) and cerebral edema (17.7% vs 8.9%, *P* = 0.031). They were also more likely to receive acyclovir (82.4% vs 68.7%, *P* = 0.009) and require ICU admission (43.9% vs 30.1%, *P* = 0.019). More ethnic minority patients presented with HIV (28.5% vs 4.5%, *P* < .001).

#### Autoimmune Subgroup

In this subgroup, more White patients presented above >60 years old (50% vs 13.2%, *P* < .001) with a CCI >2 (54.2% vs 17%, *P* < .001) and focal neurological deficits (52.1% vs 30.2%, *P* = 0.021). Ethnic minority patients were more likely to be female (67.9% vs 41.7%, *P* = 0.011), present with CSF pleocytosis (75.5% vs 44.4%, *P* = 0.002), receive vancomycin (37.7% vs 17%, *P* = 0.025) and rituximab (49.1% vs 19.1%, *P* = 0.002), require ICU admission (49.1% vs 23.4%, *P* = 0.010), and have a longer hospitalization (33 [17-54] vs 11,^6-23^ *P* < .001).

### Sex-based Subgroup Analysis

We conducted an additional subgroup analysis to assess for sex-based differences between White and ethnic minority patients regarding clinical presentation and outcomes (Supplemental Table 2). Due to the number of variables in this subgroup analysis, we only included significant variables.

#### Male Subgroup

In this subgroup, more White patients presented >60 years old (44.3% vs 27.7%, *P* = 0.003) with neck stiffness (25.4% vs 11%, *P* = 0.018), memory deficit (36.7% vs 24.5%, *P* = 0.023), and movement disorder (17.4% vs 8.1%, *P* = 0.018). Male White patients were more likely to receive IVIG (20.9% vs 10.8%, *P* = 0.018) and have a GOS <4 (59.3% vs 42.9%, *P* = 0.007). Male ethnic minority patients were more likely to have co-existing HIV (26.9% vs 4%, *P* < .001), be immunocompromised (30.9% vs 19.5%, *P* = 0.023), and present with CSF pleocytosis (87.8% vs 67.5%, *P* < .001) and a higher neutrophil count (median [IQR] 10 [0-43.25] vs 1 [0-33.5]). These patients were also more likely to have abnormal CT (41.5% vs 22.6%, *P* = 0.013) and EEG (85.9% vs 70.9%, *P* = 0.012) findings, receive vancomycin (63% vs 47.5%, *P* = 0.007), be admitted to the ICU (44.6% vs 33.3%, *P* = 0.046), and have a lengthier hospitalization (median [IQR] 14 [7-29.5] vs 10,^5-18^ *P* = 0.004).

#### Female Subgroup

In this subgroup, more White patients presented >60 years old (41.8% vs 20.3%, *P* < .001) with a CCI >2 (51.6% vs 37.8%, *P* = 0.016) and unknown etiology (47.1% vs 33.8%, *P* = 0.019). Female White patients were more likely to have a GOS <4 (61.7% vs 49.3%, *P* = 0.042). Female ethnic minority patients were more likely to have co-existing HIV (14.1% vs 2.8%, *P* < .001), have a seropositive autoimmune etiology (23.6% vs 13.7%, *P* = 0.027), and receive vancomycin (60.1% vs 46.9%, *P* = 0.023) and rituximab (12.8% vs 6.1%, *P* = 0.049).

### Comorbidity Subgroup Analysis

HIV status was significant in our initial analysis between the two groups, as was the presence of comorbidities in our etiology subgroup analysis. An additional subgroup analysis to assess the role of comorbidities in clinical outcomes and socioeconomic status demonstrated the following results.

#### Presence of Comorbidity (CCI >2) Subgroup

In this subgroup, more White patients had a GOS <4 (66.4% vs 50.9%, *P* = 0.013) and higher median household income (median [IQR] 90,468 [66,025-120,947] vs 55,842 [45,714-73,148], *P* < .001). ICU admission, length of hospitalization, mortality, and presence of insurance did not differ between the two populations (Supplemental Table 3).

#### Absence of Comorbidity (CCI ≤2) Subgroup

In this subgroup, more White patients had a GOS <4 (54.7% vs 42.4%, *P* = 0.036) and had insurance (92.1% vs 80%, *P* = 0.004), along with a higher median household income (median [IQR] 93,115 [69,559-124,933] vs 68,141 [49,237-92,624], *P* < .001). Ethnic minority patients had a lengthier hospitalization on average (median [IQR] 11^5-30^ vs 9.5 [4-18.75], *P* = 0.002). ICU admission and mortality did not differ between the two populations.

## Discussion

Our retrospective cohort study of adults with encephalitis revealed several findings: 1. White patients are more likely to present at an older age and with memory deficits, 2. Ethnic minority patients are more likely to present with coexisting HIV, abnormal EEG findings, CSF pleocytosis, receive vancomycin, and have worse outcomes, 3. Abnormal MRI and GCS <13 are independent predictors of an adverse clinical outcome, and 4. Lower socioeconomic status is associated with a higher prevalence of comorbidities and being of ethnic minority race; however, it does not significantly affect in-hospital outcomes.

Despite a robust sample size, our study revealed a significant difference in age distribution between White and ethnic minority patients with encephalitis. Notably, a significantly higher proportion of White patients presented above 60 years old, with a median of 56 years old compared to 44 in ethnic minority patients. Older age upon presentation may explain the greater proportion of White patients who presented with memory deficits. To our knowledge, there has not yet been any data revealing the substantial age gap at presentation between different racial groups in encephalitis, a disease that presents with both infectious and autoimmune etiologies. It is known that anti-NMDAR autoimmune encephalitis more commonly presents initially in younger adults,^9^ and that multiple sclerosis presents earlier with higher disease severity in Black populations.^4^ A study done in the UK in 2022 revealed that neurological autoimmune diseases like myasthenia gravis and multiple sclerosis were diagnosed earlier in mixed-race, South Asian, and African-Caribbean populations than in White populations.^21^ The authors hypothesized that increased inflammation at a younger age, psychosocial stress, socioeconomic status, and mode of onset of disease could all be potential factors contributing to an earlier onset of autoimmune disease in non-White patients, as seen in our ethnic minority patients. With regards to infectious encephalitis, a substantially higher percentage of ethnic minority patients in our study had coexisting HIV, possibly predisposing them to infection at an earlier age and playing a part in the substantial age difference at presentation.

**Figure 2.**
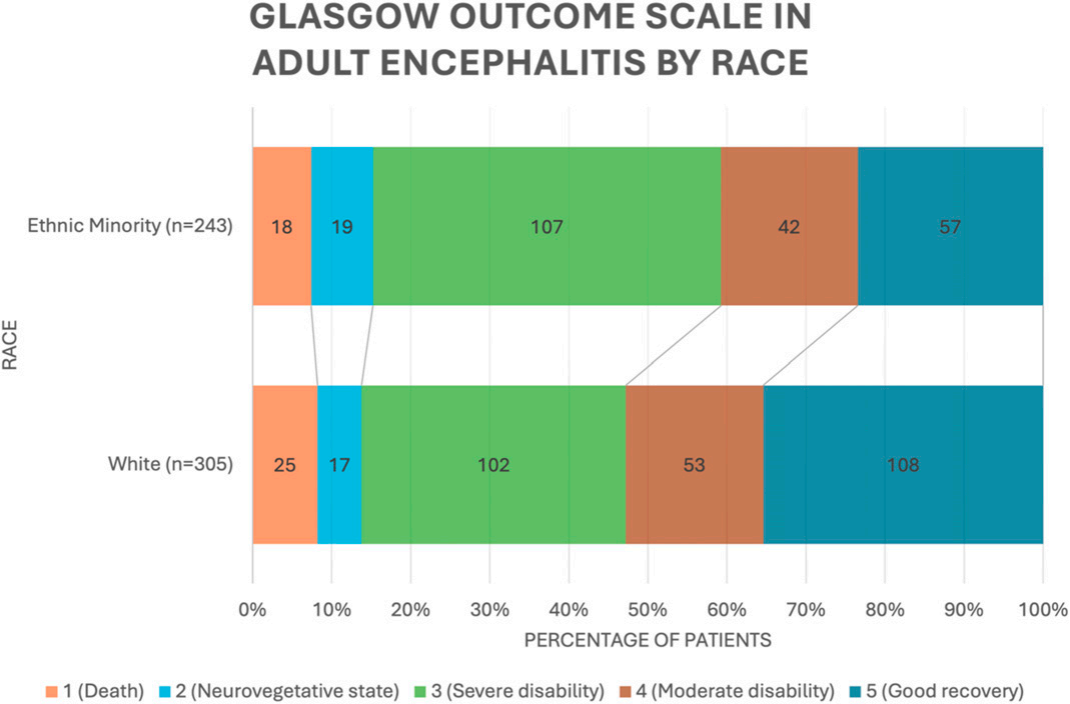
Glasgow outcome scale in adult encephalitis by race

Ethnic minority patients were more likely to present with severe organ dysfunction and/or failure, CSF pleocytosis, and abnormal EEG findings. The higher initial disease severity observed in ethnic minority patients is likely due to a complex, multifactorial etiology. Contributing factors may include underlying immunodeficiency, limited access to primary or preventative care, sociocultural barriers to timely treatment, and potential genetic predispositions to a pro-inflammatory state at a younger age.^21,22^ CSF pleocytosis has also been found to be associated with greater neurologic severity in encephalitis patients at admission.^23^ In our study, patients of an ethnic minority were more likely to receive vancomycin and have an adverse clinical outcome as defined as GOS <4, with a longer hospitalization on average. Of note, mortality rates were comparable between the two populations, suggesting that a higher proportion of ethnic minority patients were discharged with severe disability (GOS score = 3) or in a neurovegetative state (GOS score = 2) compared to White patients (Figure 2). In conjunction with the finding that ethnic minority patients presented with greater disease severity initially, it is possible to conclude that these patients were more likely to have adverse clinical outcomes due to their initial presentation, rather than any major factors during their hospitalization.

Our logistic regression analysis identified abnormal MRI as an independent predictor of an adverse clinical outcome as defined by GOS <4. In autoimmune encephalitis, MRI has been shown to have little association with outcomes as defined by Modified Rankin Scale (mRS), seizure rate, or relapse/mortality rates.^24^ However, data has shown specific MRI abnormalities to be independent predictors of adverse outcomes (mRS) in certain patient populations; eg, abnormalities in >3 lobes and left thalamic lesions in herpes simplex encephalitis^25^ and focal cortical hyperintensity in pediatric encephalitis patients.^26^ Our results suggest that further work is needed to define the prognostic value of MRI in outcomes of patients with encephalitis, with particular attention to specific MRI abnormalities and the interplay with etiology. Age over 60 years was significantly associated with GOS <4 in our initial bivariate analysis, but was found to not be an independent predictor of GOS <4 after adjustment. There are current mixed reports on whether older age is an independent predictor of adverse clinical outcomes in encephalitis,^24,27^ and our regression suggests that age does not play a substantial role in clinical outcomes in encephalitis when adjusting for other factors. GCS <13 has been previously found to be another independent predictor of an adverse clinical outcome,^27^ demonstrated by our binary logistic regression as well.

Recent research has suggested non-White ethnicity as a risk factor for NMDAR encephalitis,^8,9^ which disproportionately affects African American, Hispanic, and Asian/Pacific Islander persons in incidence and prevalence,^10,28^ though there has not yet been a study looking at race in all subtypes of autoimmune encephalitis. Our study did not find a higher incidence of seropositive autoimmune encephalitis among ethnic minority patients compared to White patients (18.5% vs 15.7%, *P* = 0.369). This may be due in part to the exclusion of at least 25 ethnic minority patients with probable and possible seronegative autoimmune encephalitis (ie, limbic encephalitis, brainstem encephalitis, Hashimoto’s encephalopathy) from the seropositive autoimmune etiology group and inclusion into the unknown etiology group. One additional factor to consider is that data was extracted from two large metropolitan areas in the U.S. with differing racial makeup, possibly contributing to further variation from previous studies.

### Socioeconomic Analysis

Public insurance, lack of health insurance, and lower median household income were associated with ethnic minority race, underlying health conditions (immune status, coexisting HIV), and infectious encephalitis. Of note, patients with autoimmune encephalitis were more likely to have private or dual insurance, and higher median household income while those with infectious encephalitis were more likely to have public or no insurance, and lower median household income. The association we found between autoimmune etiology and patients on dual insurance may be partially due to the extensive and costly workup that precedes a diagnosis of autoimmune encephalitis. A study in 2019 found that hospital charges and length of stays for patients diagnosed with autoimmune encephalitis were three to four times higher than for those with herpes simplex encephalitis, a common type of infectious encephalitis.^29^ In our study, the disparities found at initial presentation did not appear to translate into differences when comparing outcomes (ICU admission, GOS, mortality rates). The lack of substantial difference in outcome between insurance groups and income levels suggests socioeconomic status plays a role in overall health characteristics upon presentation, but that patients of different races and economic backgrounds may receive similar care during hospitalization. However, this socioeconomic analysis is limited to insurance and household income, and further analysis should include education levels, social support, food insecurity, and environmental factors.

### Subgroup Analyses

Within the etiology subgroup analysis, age >60 years was still significant within both infectious and autoimmune subgroups, demonstrating that etiology may not play a substantial role in the age of onset of encephalitis. Within the infectious subgroup, more ethnic minority patients presented with HIV, suggesting the role of immunodeficiency in predisposing patients to infectious CNS disease. More ethnic minority patients in the autoimmune subgroup were female, consistent with previous data demonstrating the prevalence of autoimmune encephalitis in females.^9,26^ Recent research has found that the CCI score was significantly higher in encephalitis patients without a viral etiology.^30^ Coupled with our finding that a CCI >2 was only significant between our two population groups within the autoimmune subgroup, this may imply an interplay between comorbidities and race in autoimmune, but not infectious disease.

The differences found between the two population groups across infectious and autoimmune encephalitis could be due to a variety of factors,^20^ including variation in initial presentation, illness severity, and disease progression between races depending on encephalitis etiology, which may lead to differences in treatment. Though there were no significant differences in the proportion of both population groups presenting with each etiology cluster (infectious, seropositive autoimmune, unknown; see Table 1), our subgroup analysis highlights the importance of considering etiology in the context of race in a patient’s disease progression.

Our sex-based subgroup analysis revealed that across both males and females, a higher proportion of White patients were >60 years old and had a GOS <4, while more ethnic minority patients had coexisting HIV and received vancomycin–findings consistent with our initial cohort analysis. Notably, male ethnic minority patients presented with more severe illness at admission, as indicated by higher rates of CSF pleocytosis and ICU admission. A recent 2023 study described sex-based differences in neurocognitive outcomes following brain inflammation and infection, noting that male patients were more susceptible to infection and associated adverse outcomes such as ICU admission.^31^ Existing literature suggests that sex hormones modulate immune response and susceptibility through the effects of estrogens, progesterone, and androgens on pro- and anti-inflammatory cytokine pathways.^32^ In addition, the relationship between blood-brain barrier (BBB) permeability and sex hormones has been established, with female sex generally associated with increased BBB integrity.^33-35^ These biological mechanisms may partially explain why male sex appeared to exacerbate the disease severity associated with ethnic minority race in our cohort.

The intersection between race and sex has been previously examined in other neuroinflammatory diseases such as multiple sclerosis, where Black women were found to have the worst clinical outcomes.^36^ In contrast, our study found greater initial disease severity among ethnic minority men. These differing sex patterns may be due to the underlying etiological distinctions of each disease, with multiple sclerosis being an autoimmune condition and encephalitis encompassing both infectious and autoimmune etiologies. The protective role of estrogen in memory function has also been reported.^37^ Our male patients did indeed have differences in presence of memory deficits, suggesting an interplay between age, race, sex, and memory. Additionally, female ethnic minority patients more frequently presented with autoimmune encephalitis, further highlighting the intersectional relationship between female sex, ethnic minority race, and autoimmune disease.^9,26^

In our comorbidity subgroup analysis, White patients were more likely to have a GOS <4 regardless of comorbidity status. While pre-existing health conditions likely influence clinical outcomes (the proportion of patients with GOS <4 was higher in both White and ethnic minority patients with comorbidities), they do not appear to completely account for differences in neurological recovery. Notably, among patients without comorbidities, there were differences in hospitalization length and insurance status between the two racial populations. This finding underscores the potential role of race in clinical outcomes even after matching patients with and without complex medical presentations. Taken together, our subgroup data strongly support that the role of racial disparities in clinical presentation and outcomes in encephalitis is complex and multifactorial.

## Strengths and Limitations

Our study contributes to a limited body of knowledge on racial and ethnic disparities in encephalitis, specifically focusing on adult patients with both infectious and autoimmune etiologies included in a larger sample size. We included robust data from two large metropolitan areas in the United States.

Although a brief subgroup analysis was conducted comparing infectious and autoimmune encephalitis between our two groups, the primary focus of this study was all-cause encephalitis. A more comprehensive stratified analysis by etiology is needed in future research to better elucidate etiology-specific disparities. Additionally, patients were initially dichotomized into White and ethnic minority patients to assess broad differences; subsequent analyses will examine outcomes by individual racial and ethnic populations, including Black, Hispanic, and Asian patients. We acknowledge that grouping Black, Hispanic, and Asian patients into the ‘Ethnic minority’ population group does not account for the genetic, environmental, and socioeconomic differences between the groups.

This study was restricted to the data included in the electronic medical record due to its retrospective design. As a result, some information was missing or unknown, which resulted in a less robust sample for certain variables. The retrospective design also limited the ability to assess whether access to healthcare services was standardized across all patients, as differences in the quality and/or availability of diagnostic modalities and treatments were not readily obtainable in patients’ charts. Only patients with seropositive CSF results were classified as having an autoimmune etiology, leaving a sizable portion of patients with probable or possible seronegative autoimmune encephalitis in the unknown etiology group. The large proportion of patients with unknown etiologies upon admission suggest that encephalitis, particularly autoimmune, may be underdiagnosed or diagnosed belatedly. Though the study included data from two substantial metropolitan areas in the United States, the results may not be generalizable to less populated areas or other countries at this time. The socioeconomic analysis was limited by the fact that median household income was determined based on patient zip code, and thus was an estimate of the patient’s income per zip code area.

## Conclusion

Ethnic minority patients with encephalitis tend to present at a younger age and are more likely to have co-existing HIV, severe initial organ dysfunction, CSF pleocytosis, abnormal EEG findings, and worse clinical outcomes. These findings highlight the potential role of racial disparities in contributing to disease severity and abnormal diagnostic findings at presentation, possibly leading to unfavorable clinical outcomes. Binary logistic regression identified abnormal MRI and GCS <13 upon presentation as independent predictors of a poor clinical outcome–prognostic markers that, with further validation, may help guide risk stratification and prognosis during hospitalization. Clinical outcomes did not differ between patients with varying household income levels and insurance types, suggesting excellence in equitable care across adult encephalitis patients in these two metropolitan areas.

## Supplemental Material

**Supplemental Material -** Racial and Ethnic Disparities in Clinical Characteristics and Outcomes in Adults With Encephalitis: A Retrospective Study Supplemental Material for Racial and Ethnic Disparities in Clinical Characteristics and Outcomes in Adults With Encephalitis: A Retrospective Study by Sienna Wu, Rodrigo Hasbun, Ralph Habis, Jordan Benderoth, Ivany Patel, Ashutosh Gupta, Megan Goyal, Arun Venkatesan, John C. Probasco, Paris Bean, Ashley Heck, Laya Rao, Rajesh Gupta in Journal of Central Nervous System Disease.

## Data Availability

Anonymized data utilized for this study will be made available upon reasonable request to the corresponding author.*
