# Supplementary material for: Racial and Ethnic Disparities in Clinical Characteristics and Outcomes in Adults With Encephalitis: A Retrospective Study
Source: J Cent Nerv Syst Dis. 2026 Jan 15;18:11795735251414833. doi: 10.1177/11795735251414833 (PMC12811602; doi:10.1177/11795735251414833)
Supplement: Supplemental Material - Racial and Ethnic Disparities in Clinical Characteristics and Outcomes in Adults With Encephalitis: A Retrospective Study [file sj-pdf-1-cns-10.1177_11795735251414833.pdf]

Supplemental Tables

Table 1. Socioeconomic Factors Analysis, as defined by Median Household Income

**Clinical Feature** n/N (%)		**Median Household Income** (Median)	**P Value**[a]
Age	<60 years	74,466	0.145
	>60 years	82.300	
**Race**	**Non-Hispanic White**	**90,772**	**<.001**
	**Ethnic Minority**	**59,224**	
**Immune Status**	**Immunocompetent**	**80,335**	**0.001**
	**Immunocompromised**	**68,962**	
**HIV Status**	**Negative**	**80,692**	**<.001**
	**Positive**	**50,284**	
CCI	<2	78,864	0.217
	>2	73,579	
**Infectious**	**No**	**80,122**	**<.001**
	**Yes**	**71,609**	
**Autoimmune**	**No**	**74,707**	**<.001**
	**Yes**	**92,722**	
ICU Admission	No	78,424	0.095
	Yes	72,337	
Mortality	Alive	77,694	0.959
	Deceased	79,446	
GOS	>4	83,627	0.213
	<4	75,479	

Abbreviations: HIV, Human Immunodeficiency Virus; CCI, Charlson Comorbidity Index; ICU, Intensive Care Unit; GOS, Glasgow Coma Scale
[a]P-value for difference between different income levels

Table 2. Clinical Presentation and Outcomes in Adult Encephalitis Stratified by Sex, Significant Variables

**Clinical Feature**<br>n/N (%)	**White**	**Ethnic Minority**	**P Value**[a]
Male sex (n=298)			
Age, median (IQR)	57 (41-67)	44 (31-61)	<.001
Age >60 years	70/158 (44.3)	38/137 (27.7)	0.003
Presence of HIV	6/151 (4)	36/134 (26.9)	<.001
Immunocompromised	31/159 (19.5)	43/139 (30.9)	0.023
Neck stiffness	15/59 (25.4)	11/100 (11)	0.018
Memory deficit	58/158 (36.7)	34/139 (24.5)	0.023
Movement disorder	27/155 (17.4)	11/136 (8.1)	0.018
CSF pleocytosis[b]	104/154 (67.5)	122/139 (87.8)	<.001
CSF neutrophil, median (IQR)	1 (0-33.5)	10 (0-43.25)	0.006
Abnormal CT findings	14/62 (22.6)	44/106 (41.5)	0.013
Abnormal EEG findings	83/117 (70.9)	73/85 (85.9)	0.012
Vancomycin	75/158 (47.5)	87/138 (63)	0.007
IVIG	33/158 (20.9)	15/139 (10.8)	0.018
ICU Admission	53/159 (33.3)	62/139 (44.6)	0.046
Length of stay in days, median (IQR)	10 (5-18)	14 (7-29.5)	0.004
GOS <4	83/140 (59.3)	57/133 (42.9)	0.007
Female sex (n=301)			
Age, median (IQR)	56 (41.5-67)	40.5 (29.25-56)	<.001
Age >60 years	64/153 (41.8)	30/148 (20.3)	<.001
Charlson Comorbidity Index >2	79/153 (51.6)	56/148 (37.8)	0.016
Presence of HIV	4/142 (2.8)	20/142 (14.1)	<.001
Seropositive autoimmune etiology	21/153 (13.7)	35/148 (23.6)	0.027
Unknown etiology	72/153 (47.1)	50/148 (33.8)	0.019
Vancomycin	69/147 (46.9)	89/148 (60.1)	0.023
Rituximab	9/147 (6.1)	19/148 (12.8)	0.049
GOS <4	82/133 (61.7)	66/134 (49.3)	0.042

Abbreviations: IQR, Interquartile Range, HIV, Human Immunodeficiency Virus; CSF, Cerebrospinal Fluid; CT, Computed Tomography; EEG, Electroencephalogram; IVIG, Intravenous Immunoglobulin; ICU, Intensive Care Unit; GOS, Glasgow Outcome Scale

[a]P-value for difference between White and ethnic minority patients

[b]White blood cell count ≥5 cells/μL

Table 3. Clinical Outcomes and Socioeconomic Status in Adult Encephalitis Stratified by Presence of Comorbidities

**Clinical Feature**<br>n/N (%)	**White**	**Ethnic Minority**	**P Value**[a]
CCI >2 (n=281)			
ICU admission	63/152 (41.4)	56/123 (45.5)	0.497
Length of stay in days, median (IQR)	12 (7-22)	12 (8-25)	0.722
**GOS <4**	**89/134 (66.4)**	**59/116 (50.9)**	**0.013**
Mortality	12/157 (7.6)	11/124 (8.9)	0.709
**Household income, median (IQR)**	**90,468 (66,025-120,947)**	**55,842 (45,714-73,148)**	**<.001**
Has insurance	133/141 (94.3)	102/115 (88.7)	0.102
CCI ≤2 (n=318)			
ICU admission	50/152 (32.9)	61/163 (37.4)	0.400
**Length of stay in days, median (IQR)**	**9.5 (4-18.75)**	**11 (5-30)**	**0.002**
**GOS <4**	**76/139 (54.7)**	**64/151 (42.4)**	**0.036**
Mortality	13/155 (8.4)	7/163 (4.3)	0.133
**Household income, median (IQR)**	**93,115 (69,559-124,933)**	**68,141 (49,237-92,624)**	**<.001**
**Has insurance**	**117/127 (92.1)**	**119/149 (80)**	**0.004**

Abbreviations: CCI; Charlson Comorbidity Index; ICU; Intensive Care Unit; IQR, Interquartile Range; GOS, Glasgow Outcome Scale

[a]P-value for difference between White and ethnic minority patients
